# The role of attention in the effect of facial attractiveness on time perception

**DOI:** 10.1002/pchj.744

**Published:** 2024-03-26

**Authors:** Weiwen Wu, Yu Tian

**Affiliations:** ^1^ Institute of Brain and Psychological Sciences Sichuan Normal University Chengdu China

**Keywords:** attention, attractiveness, dot‐probe task, dual‐task, time perception

## Abstract

Recent research has indicated that attractive faces often cause a dilation of our time perception thus affecting physical and mental health, and speculates that this could be relevant to the fact that attractive faces capture people's attention. Nevertheless, there was no direct experimental data to support this speculation. The present work was designed to illustrate how attention affects time perception of facial attractiveness. It utilized two experiments to investigate this phenomenon. In Experiment 1, perception of timing and attention bias were assessed using a temporal reproduction task and a dot‐probe task. Increased attention bias was found to mediate the time dilation effect of facial attractiveness. Experiment 2 adopted dual‐task paradigm, combining a temporal reproduction task and attractiveness rating task, to manipulate attention allocation. The findings suggested that allocating more attention to the task requiring timing enhanced the time dilation effect caused by the faces. Results of Experiments 1 and 2 converge to show that attention plays an essential role in the effects of facial attractiveness on time perception.

## BACKGROUND

Albert Einstein said, “A minute with your hand on a hot stove feels like an hour. An hour sitting with a beautiful girl feels like a minute.” By comparing experience of keeping one hand over the hot fireplace to spending time with an attractive companion, Einstein highlights how our perception of timing can vary based on our level of engagement and the intensity of our experiences. This quote is often used to illustrate the idea that time can feel either elongated or compressed depending on our subjective experience, emphasizing the relative nature of our perception of timing. Previous research has demonstrated that facial attractiveness can impact perception of timing, leading to longer subjective reproduced durations (Ogden, 2013; Tian et al., 2019; Tomas & Španić, 2016), whereas Einstein argued that facial attractiveness shortens the perceived durations of time. The paradoxical relationship is most likely to be the result of attractive faces capturing a large amount of attentional resources, which leads to a decrease in attention allocated to timing. Therefore, the key to explaining the difference between actual time and subjective time perception and the factors influencing it lies in the attentional mechanism (Liu et al., 2021).

The effect of attention in the area of psychology that specializes in the study of time perception has been extensively investigated. Initially, the changes in perception of timing could be understood by the pacemaker–accumulator (PA) model (Gibbon et al., 1984; Treisman, 1963). The hypothesis of the model is that perception of timing is generated by the pacemaker, switch/gate, and accumulator. Specifically, during completing the temporal task, the pacemaker sends out pulses that reach the accumulator through the switch/gate, and the final accumulated number of pulses on behalf of perceived time. Similarly, the scalar expectancy theory was expanded as an extension of the PA model to further understand the mechanisms underlying perception of timing. It divides the timing processing into three phases, that is, the clock, the memory, and the judgement phase. According to this model, the direction and degree of distortion of subjective time perception depends on which phase of the model is affected. It is generally believed that an increase in arousal during the clock phase causes the pacemaker to send pulses at a higher frequency and generating more pulses, thus leading to an overestimation of time perception; in addition, closing the attention‐controlled switch that makes it possible for the pacemaker to send increased pulses to the accumulator, it also results in a dilation of subjective perception of timing. Zakay and Block (1997) introduced the attentional‐gate model (AGM) as a theoretical framework that provides insights into how attention affects perceived time. In the AGM, concepts of the time information processing switch were replaced by the attention gate. Unlike a switch that operates in an “all” or “none” manner, the degree to which the attentional gate is opened or closed depends on how much attention is given to timing. When attentional resources are directed towards timing, the more open the attentional gates are, the more impulses pass through, leading to a longer subjective perception of timing. There is evidence that highly arousing stimuli can lead to temporal overestimation, while a lack of attention to timing can lead to temporal underestimation (Zakay & Block, 1997).

Attractive faces have a number of features that can be pleasurable as a source of aesthetic stimuli, and are often accompanied by a certain physiological arousal, as well as a certain proximity motivation that is often induced (O'Doherty et al., 2003). According to existing studies, it is known that attractive faces enhance arousal and attract more attention, thus affecting time perception (Nakamura & Kawabata, 2014; Ren et al., 2021; Zhou et al., 2021). According to existing models of time perception, attention and arousal are two important factors that influence time perception. Previous research has extensively elaborated the impact of arousal on subjective perception of timing, particularly how a change in arousal levels would influence it (Droit‐Volet et al., 2020; Piovesan et al., 2019; Tian et al., 2019; Tomas & Španić, 2016). Furthermore, Zhou et al. (2021) found that enhanced arousal plays a mediating role of the time dilation effect induced by the attractiveness of a face. However, the precise effect of attention in the link between facial attractiveness and perception of timing remains uncertain and requires further investigation. So, it follows that the present research attempted to delve into how attention works in the relationship between facial attractiveness and subjective perception of timing in depth on the basis of controlling the allocation of attention.

Until now, many researchers have explored the moderating role of facial attractiveness on attentional orienting, maintenance, disengagement, or transfer using behavioral tasks, such as the dot‐probe task, spatial attention task, and rapid series visual presentation task (Lindell & Lindell, 2014; Maner et al., 2007; Sui & Liu, 2009). In particular, the more widely available task measuring attention bias to stimuli is the dot‐probe task (Wirth & Wentura, 2020). Two stimuli can be presented at the same time by dot‐probe task; it enables selective attention to be measured and also reflects exactly which attentional components correspond to the attention bias, of which the attention bias is an important indicator to investigate the selective bias of individuals to attend to visual stimuli for attentional processing (Maner et al., 2007). However, theoretical explanations of attentional mechanisms by which facial attractiveness affects perception of timing still lack direct empirical support (Martinelli & Droit‐Volet, 2022; Tian et al., 2019). According to the scalar expectancy theory, the earlier attention is captured, the earlier the switch is subsequently closed, so that more pulses reach the accumulator through the switch, causing a time dilation effect (Gibbon et al., 1984). Therefore, we proposed the following hypothesis:

**Hypothesis 1:** Attractive faces elicit more attention bias more quickly, which affects the time perception of faces, that is, attention bias appears to play a mediating role for the impact of facial attractiveness on subjective perception of timing.

In Experiment 1, attention bias towards appealing faces was investigated using a dot‐probe task. Additionally, a reproduction task was employed to obtain the reproduced durations of attractive faces, average faces, and neutral images. A mediated effects analysis was also used to examine Hypothesis 1, that increased attention bias mediates the temporal overestimation caused by attractiveness of faces.

In addition, several studies have investigated the dynamics of attentional manipulation during perception of timing through a dual‐task paradigm, showing that subjective temporal intervals are prolonged when attention is allocated on the temporal task, and shortened when attention is allocated to another task unrelated to timing (Chen et al., 2007). However, could these findings provide an explanation for the role of attention during the process by which facial attractiveness affects the perception of timing? Further proof is needed. Time perception experiments are generally conducted by using either dual or single task condition. The dual‐task condition mainly requires participants to complete a temporal task while performing a task that was not related to timing. The single‐task condition mostly uses the odd‐ball paradigm (Lin & Qian, 2013). In particular, many researchers have demonstrated through the dual‐task paradigm that processing time requires attentional engagement (Hallez & Droit‐Volet, 2019). According to the AGM, increased allocation of attention to temporal task results in greater opening of the attentional gates, allowing a larger count of pulses to cross and reach the accumulator. This, in turn, leads to overestimation in time perception (Zakay & Block, 1997), and the dual‐task paradigm enables effective modulation of attention allocation to explore the influence of different levels in attention on perception of timing. Therefore, we proposed the following hypothesis:

**Hypothesis 2:** When more attention is allocated to temporal task, the time dilation effect induced by facial attractiveness is significantly enhanced.

Experiment 2 attempted to manipulate the allocation of attention through a dual‐task paradigm (including an attractiveness rating task and a temporal reproduction task), using attractive faces, average faces as experimental material, and recording participants' ratings and reproduced durations of images at different levels of attention.

Consequently, this study aimed to achieve two main objectives. First, it aimed to assess attention bias towards faces by employing a dot‐probe task, and further exploring what mediates the role of attention bias in the perception of timing during facial attractiveness (Experiment 1). Second, Experiment 2 utilized a dual‐task paradigm to manipulate attentional allocation and investigate the relationship between attention and time overestimation phenomenon. Additionally, participants' subjective time feelings were evaluated through a temporal reproduction task.

## EXPERIMENT 1

### Method

#### Participants

The calculation of the number of participants needed was determined based on previous research using the reproduction task (Zhou et al., 2021), and analysis with G*Power. It was found that 40 participants were needed to satisfy the 80% power to test the time dilation effect caused by facial attractiveness (Faul et al., 2007).

Both physical sex and psychological gender affect the understanding and judgment of facial attractiveness (Amezcua‐Gutiérrez et al., 2020; Oh et al., 2020; Samson & Janssen, 2014; Tian et al., 2019). Also, previous studies have noted that gender moderates the effects of facial attractiveness on attention and time perception (Mitrovic et al., 2018; Tian et al., 2019), and women are more emotionally susceptible than men (Yuan et al., 2009). This may lead to other emotional variables (e.g., jealousy, admiration) in female participants when observing faces. Therefore, only male participants were used in this study. Female faces were chosen as the experimental material for this study and participants were selectively recruited (i.e., heterosexual males). Ultimately, 40 healthy males were recruited from the local university and they all reported themselves as being Chinese, heterosexual, having good vision or good vision with glasses on, and having no record of neurological or mental disorders. In addition, their ages ranged across 18 to 24 (*M* ± *SD* = 20.47 ± 1.91) years. These participants voluntarily and knowingly signed a consent form and received moderate payment.

#### Stimuli

Sixteen color images of Chinese women's faces were used as experimental materials. These faces were facing the camera, with eyes looking straight at the camera, neutral expressions, and no additional decoration in each image. The images were normalized to a 320 × 400‐pixel white background without standardization of skin color or patches, as they were considered components of attractiveness. The experimenter selected 17 color images (eight attractive female faces, eight average attractive female faces, and one neutral stimulus picture) for Experiment 1. The neutral stimulus picture was a pink ellipse image on a white backdrop. To validate the manipulation of the stimuli, in the rating section of the experimental material, a group of participants was recruited and rated how attractive and distinctive these faces were on a 9‐point scale (as shown in Table 1). Participants in the face image ratings were all male and were otherwise homogeneous with those recruited in the experiment.

**TABLE 1:** Mean (*SD*) of attractiveness and distinctiveness for the images in Experiment 1.

	Attractive face	Average face	Neutral image
Attractiveness rating	7.24 (0.41)	4.91 (0.23)	2.40 (0.29)
Distinctiveness rating	6.08 (0.48)	2.21 (0.22)	2.01 (0.35)

*Note*: Attractiveness: from 1 = *exceedingly unattractive* to 9 = *exceedingly attractive*. Distinctiveness: from 1 = *completely not distinctive* to 9 = *exceedingly distinctive*.

Analysis of variance (ANOVA) was undertaken to analyze the attractiveness ratings with stimulus type as the independent variable (attractive, average, and neutral stimulus). The findings revealed that the attractiveness scores for the three stimulus types differed significantly, *F*(1,29) = 2940.60, *p* < .001, *η*_*p*_^2^ = .99. Then, a post hoc test for Bonferroni calibration indicated that appealing faces received significantly higher attractiveness ratings compared to average (*p* < .001), while average faces received significantly higher attractiveness scores versus the neutral stimulus (*p* < .001). Similarly, an ANOVA was conducted to analyze the distinctiveness ratings with stimulus type as the independent variable. The outcome demonstrated that there was a significant difference in distinctiveness ratings among the three stimulus types, *F*(1,29) = 1637.85, *p* < .001, *η*_*p*_^2^ = .98. A post hoc test corrected for Bonferroni found that attractive faces were more distinctive than the average faces(*p*s < .001). Also, participants perceived average faces as more distinctive compared to neutral stimuli (*p* = .047).

#### Procedure

The experimenter prepared a quiet room where the experiment would take place. Each participant was tested individually. The experimental setup consisted of a 17‐inch LED monitor. The visual angles for both horizontal and vertical directions were less than 16 degrees. To provide their responses, participants used the keyboard. Experiment 1 consisted of two sections: a reproduction task and a dot‐probe task (same side). The order of the two tasks was counterbalanced between participants.

Tran et al. (2013) highlighted that attentional response displayed in the dot‐probe task could be calculated. The dot‐probe task consists of two kinds of experiment trials: congruent trials (C trials) and incongruent trials (I trials), depending on whether the target stimulus is on the same side as the probe or not. The attention bias index (BI) is obtained by subtracting the time of response for I trial (RTI) from the time of response for C trial (RTC). BI reflects the participant's attention bias towards the target stimulus, where a BI greater than 0 indicates attention bias, a BI equal to 0 indicates no bias or avoidance, and a BI less than 0 indicates attentional avoidance (Tran et al., 2013).

During the reproduction task, a fixed cross‐shaped gaze point is displayed at the start of each trial, with a presentation time of 500–750 ms, and a face stimulus presenting 1000, 2000, or 3000 ms next. Then, a “?” is displayed on the screen, prompting participants to reproduce the perceived stimulus duration. The “?” stays there for 3000 ms on the display unless the participant reacts with a press of the space bar. After pressing the key, a pink ellipse image with a white backdrop is displayed on screen till the participant lets go of the space bar. The subjective duration of the replication here corresponds to the objective duration of the face stimulus. Finally, a blank page is shown for 1000 ms, making up around 80% of the trial duration (Zhou et al., 2021). Each image was repeatedly presented eight times at 2000 ms and just one time at 1000 ms and 3000 ms, for a total of 160 trials. All trials were randomized in order across different participants.

**FIGURE 1:**
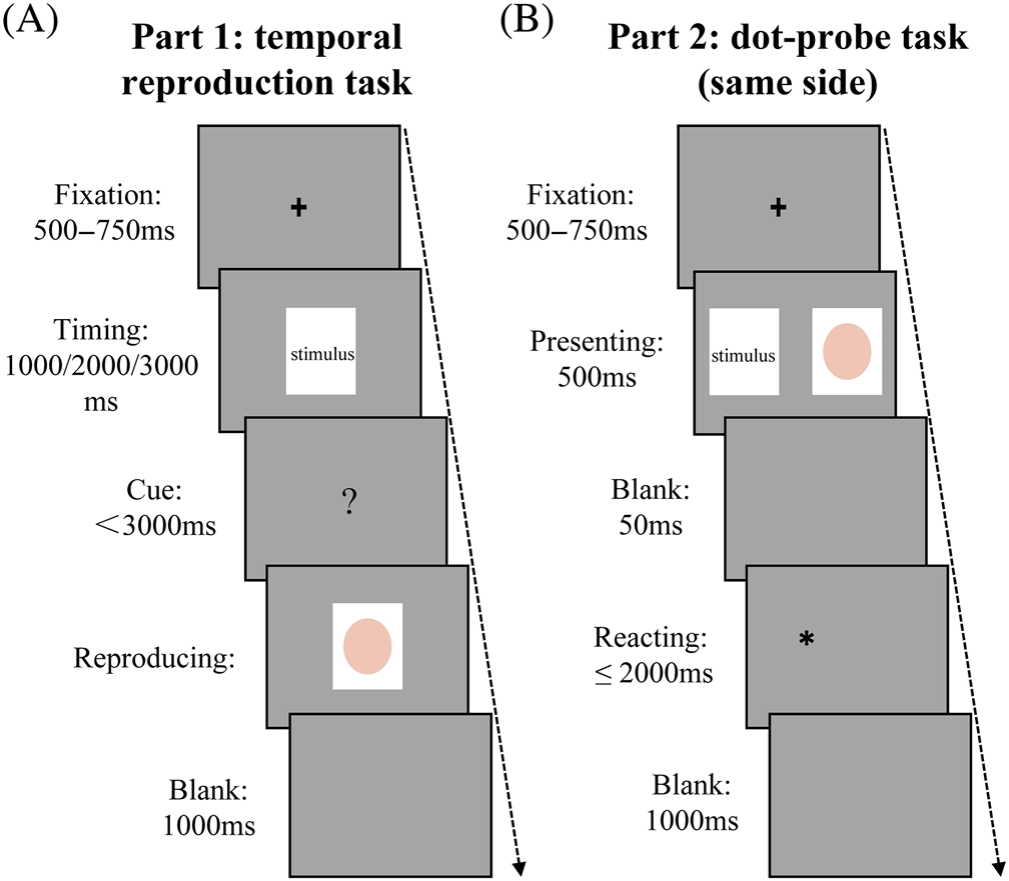
The procedure of Experiment 1. (A) Schematic illustration of the temporal reproduction task. (B) Schematic illustration of the dot‐probe task. To protect privacy, the face in the schematic is replaced by the text‐stimulus. Participants were asked to press “F” if “*” was on the left or “J” if “*” was on the right.

On the dot‐probe task (same side), the fixed point of the cross shape is first presented in the middle of the display, with a presentation time of 500–750 ms. Then, “Attractive face–Neutral stimulus” and “Average face–Neutral stimulus” image pairs were displayed on two sides of the left and right of the fixed point, with image pair positions counterbalanced across all trials. The distance between the two images presented and the central fixed point was equal. Their rendering time was 500 ms. As the image pair disappeared, a “*” occurred in the position of the facial picture (consistent condition) or the neutral stimulus (inconsistent condition), lasting for less than 2000 ms. Each participant was required to react rapidly and precisely to the position of the dot probe by pressing “F” if the “*” was at left and “J” if the “*” was at right. After pressing the key, the dot‐probe disappeared, a blank screen of 1000 ms appeared, and then participants entered the next trial; if there was no response from the participant within a specified time window, a blank page was displayed for 1000 ms to enter the next trial (see Figure 1 for the process).

### Results

#### Attractiveness of face effects on the perception of timing

A paired‐sample *t*‐test of reproduced durations for stimulus type (two levels: attractive, average) indicated a significant difference in reproduced durations for attractive faces and average faces, *t*(39) = 23.09, *p* < .001. Reproduction time of attractive faces was longer than average.

**FIGURE 2:**
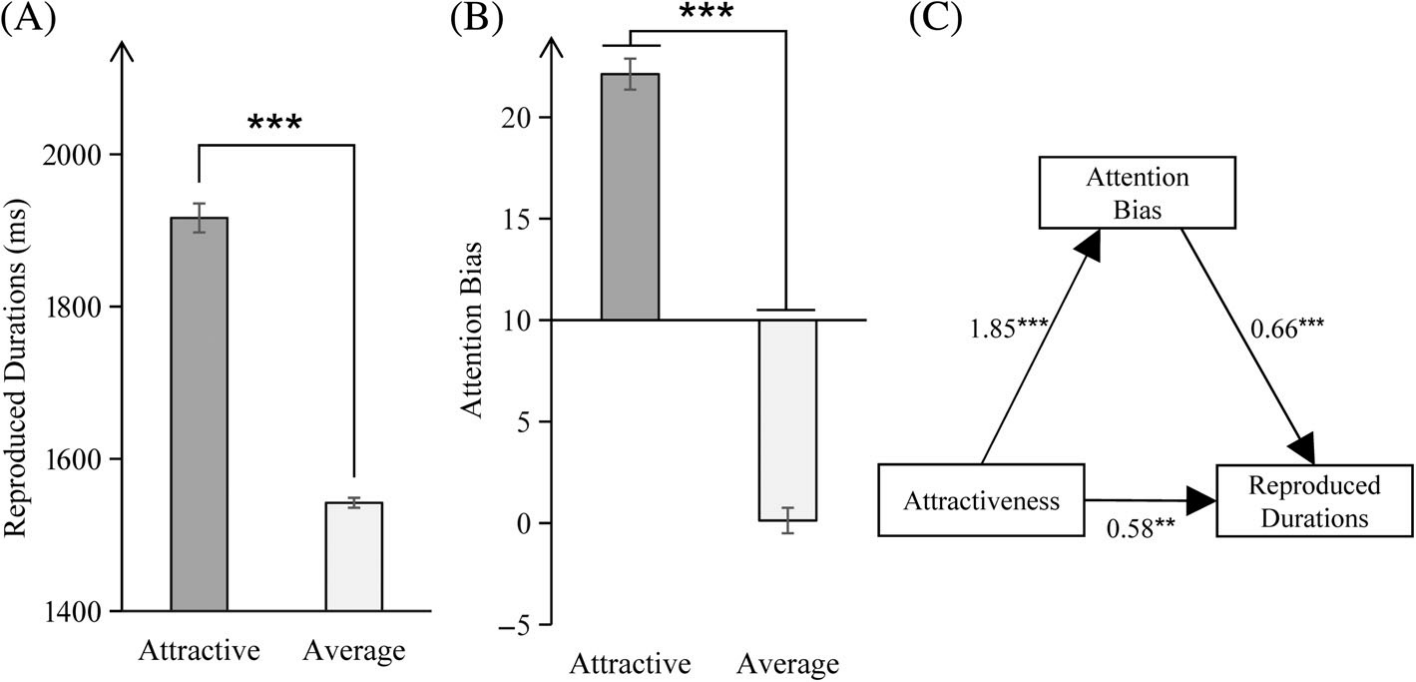
(A) Schematic representation of reproduced durations for different stimulus types. (B) Schematic representation of the attentional bias for different stimulus types. (C) Mediation model in Experiment 1. The values represent the standardized regression coefficients (*β*) for the direct and indirect effects of attractiveness on reproduced durations. Significance level ***p < .001, **p < .05.

The findings suggested that facial attractiveness has the ability to influence perception of timing. Specifically, the presence of attractive faces often led to a significant time dilation effect (refer to Figure 2A).

#### The impact of facial attractiveness on attention

A paired‐sample *t*‐test for attention bias for stimulus type (two levels: attractive, average) showed a significant difference in attention bias between attractive and average faces, *t*(39) = 22.33, *p* < .001. Attractive faces (*M* ± *SD* = 22.12 ± 4.83) produced more attention bias than average faces (*M* ± *SD* = 0.13 ± 3.98).

These findings demonstrated that the increase of facial attractiveness led to an enhancement of attention bias (see Figure 2B).

#### The mediating effect of attention in the time dilation effect produced by faces

Mediation analysis was carried out using PROCESS (Hayes, 2013) in order to investigate whether attention bias (M = bias index, BI = RTI – RTC) mediates the process by which stimulus type (X = facial attractiveness, attractive vs. average) affects time perception (Y = duration of reproduction). It was shown that attractiveness of face had a significant and direct effect on perceived time (*β* = −1.80, *p* < .001), attractiveness of face exerts a significant effect on attention bias (*β* = −1.85, *p* < .001), and there was a significant effect of attention bias on perception of timing with a significant mediating effect (*β* = −0.58, *p* = .0069), indicated a partial mediation of attention bias between facial attractiveness and perception of timing, with the mediating effect accounting for 67.95% of the total effect.

Such findings strongly underline the important mediating role of attention bias during the process by which facial attractiveness affects perception of timing. The observed increase in attention bias toward faces is indicative of a greater allocation of attentional resources toward attractive faces. This suggests that enhanced attention is a potential mechanism by which facial attractiveness induces a time dilation effect (see Figure 2C).

### Discussion

In Experiment 1, we tested whether attention mediated the time dilation effect for facial attractiveness using a dot‐probe task and a temporal reproduction task. We first reproduced the result that facial attractiveness has a time dilation effect, which was elaborated using attractive faces versus average faces as materials for the temporal reproduction task. We also discovered that attractive faces elicited significantly more attention bias than average faces, that is, attractive faces attracted more attention than average faces, which is consistent with previous findings (Chen et al., 2012; Sui & Liu, 2009; Van Hooff et al., 2011). Based on this result, we utilized the dot‐probe task to explore how attention mediates the influence of faces on the time dilation effect and used the attention bias index as a quantitative indicator of attention.

The results suggest that attention plays a very important mediation role in the effect of attractive faces on perception of timing, which supports the previous findings and the scalar expectancy theory. It is once again shown that the important mechanism by which facial attractiveness induces time dilation effect is the attention mechanism (Ogden, 2013).

However, we needed a more direct test of this potential mechanism. Therefore, in Experiment 2, we sought to adjust the level of attention allocation by means of a dual‐task paradigm (Hallez & Droit‐Volet, 2019; Macar et al., 1994) to test whether changes in attentional resource allocation would lead to changes in time perception.

## EXPERIMENT 2

### Method

#### Participants

The number‐of‐participants calculation was determined based on previous research using the reproduction task (Zhou et al., 2021), and an analysis with G*Power. It was found that 40 participants were required to satisfy the 80% power to test the time dilation effect caused by attractiveness of face.

Forty male participants aged 18 to 26 (*M* ± *SD* = 21.48 ± 2.03) years from one of China's universities participated in this experiment. They were selected based on physical health, good eyesight or good vision with eye wear, and habitual right‐handedness. All participants confirmed their heterosexual orientation and had no previous neurological or psychiatric disorders. Prior to their participation, each participant gave informed consent and received moderate payment.

#### Stimuli

Sixteen images (eight attractive and eight average faces) from Experiment 1 were chosen as the stimulus materials for Experiment 2.

#### Procedure

The experimental apparatus for Experiment 2 remained consistent with Experiment 1. Experiment 2 comprised two main components: a temporal reproduction task and an attractiveness rating task. Attentional cues were presented at the beginning of the experiment and required participants to allocate their attention to the time of stimulus presentation, or to the attractiveness of the face, or to both.

In the temporal reproduction task, a fixed cross‐shaped gaze point is displayed at the start of each trial, with a presentation time of 500–750 ms, a face stimulus presenting 1000, 2000, or 3000 ms next afterwards. After the face stimulus, a letter “T” appears on the screen, prompting participants to complete the reproduction task (i.e., duration of reproduced face images). When the “T” disappears, an ellipse image in pink on a white ground appears on the screen and participants begin to reproduce its perceived duration of facial images by holding down a space bar. The duration of holding the space bar was equivalent to their perceived duration of the face stimulus. Finally, a 1000‐ms blank screen is displayed. The duration of the target stimuli takes up approximately 80% of the total trial duration (Li et al., 2017, 2019).

**FIGURE 3:**
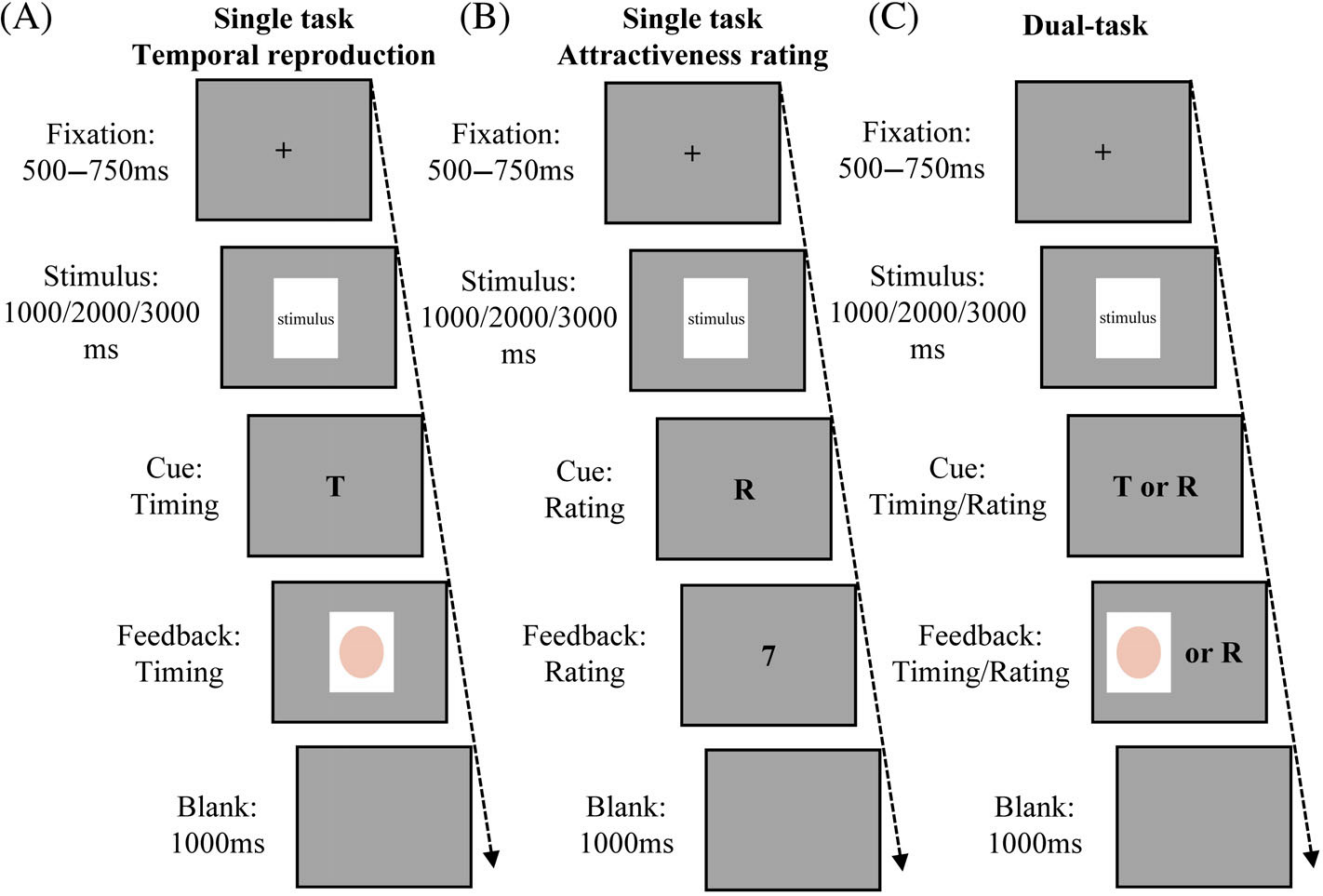
The procedure of Experiment 2. (A) Schematic illustrations of the single‐task temporal reproduction. (B) Schematic illustrations of the single‐task attractiveness rating (here is an example of Rating 7). (C) Schematic illustrations of the dual‐task paradigm.

During the attractiveness‐rating task, participants are asked to rate the attractiveness of the various images on a scale of 9 points. A fixed cross is presented at the initiation by each trial and lasts 500–750 ms, then the image of face is presented for 1000, 2000, or 3000 ms. Next, the letter “R” appears on the screen, which reminds participants that they should complete the attractiveness rating task (i.e., rating the attractiveness of the face image just presented). When the “R” disappears, a scale of ratings (from 1 = *exceedingly unattractive* to 9 = *exceedingly attractive*) appears and remains until the participant presses the keyboard to rate. Lastly, a blank screen is shown for 1000 ms(see Figure 3 for the process).

**FIGURE 4:**
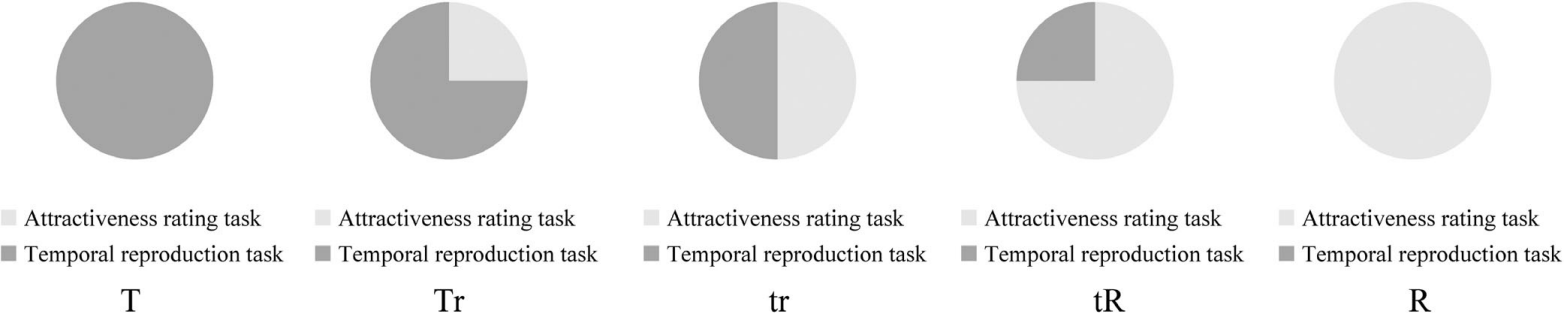
Schematic illustration of the attention to allocation instructions of Experiment 2. In the T condition, 100% of responses were to the temporal reproduction task; in the Tr condition, 75% of responses were to the temporal reproduction task and 25% were to the attractiveness rating task; in the tr condition, 50% of responses were to the temporal reproduction task and 50% were to the attractiveness rating task; in the tR condition, 25% of responses were to the temporal reproduction task and 75% were to the attractiveness rating task; in the R condition 100% of the responses were to the attractiveness rating task.

Figure 4 illustrates the allocation of attentional resources across the reproduction task and the attractiveness rating task in Experiment 2. The dark gray bar represents the attention assigned to the reproduction task and the light gray bar represents the rating task. We asked participants to allocate their attentional resources to the two tasks according to five different allocation ratios as instructed, as shown in Figure 4.

There were 160 trials in the Experiment 2, with 32 trials in each of the conditions according to the allocation of different proportions of attentional resources. Order effects were avoided by a Latin square design, so that trial order was randomized across participants.

In order to be familiarized with the experimental routine, all participants practiced prior to the experiment, which was identical to the formal experimental procedure. During practice, participants learned and mastered the requirement of distributing attention as shown in Figure 4. Instructions and attentional cues were presented to participants. Each participant made sure they knew this procedure and the meanings of the attentional allocation reminders before starting the practice. In Experiment 2, we recorded the results of reproduced durations and attractiveness ratings of participants. The temporal reproduction task included four attentional allocation conditions (T, Tr, tr, and tR) and the attractiveness rating task included four attentional allocation conditions (R, tR, tr, and Tr).

Following the finish of Experiment 2, we tested whether participants had allocated their attentional resources to the reproduction task and the rating task according to the condition requirements via a scale of 9 points (from 1 = *completely do not understand* to 9 = *understand perfectly*). Just three participants selected a score of 8, and the remaining participants selected a score of 9. The results of the Guidance Adherence Rating (*M* ± *SD* = 8.93 ± 0.27) showed that all participants perceived their attentional resources to be allocated exactly as instructed. Therefore, no participant failed to perform the experimental manipulation as required.

### Results

**FIGURE 5:**
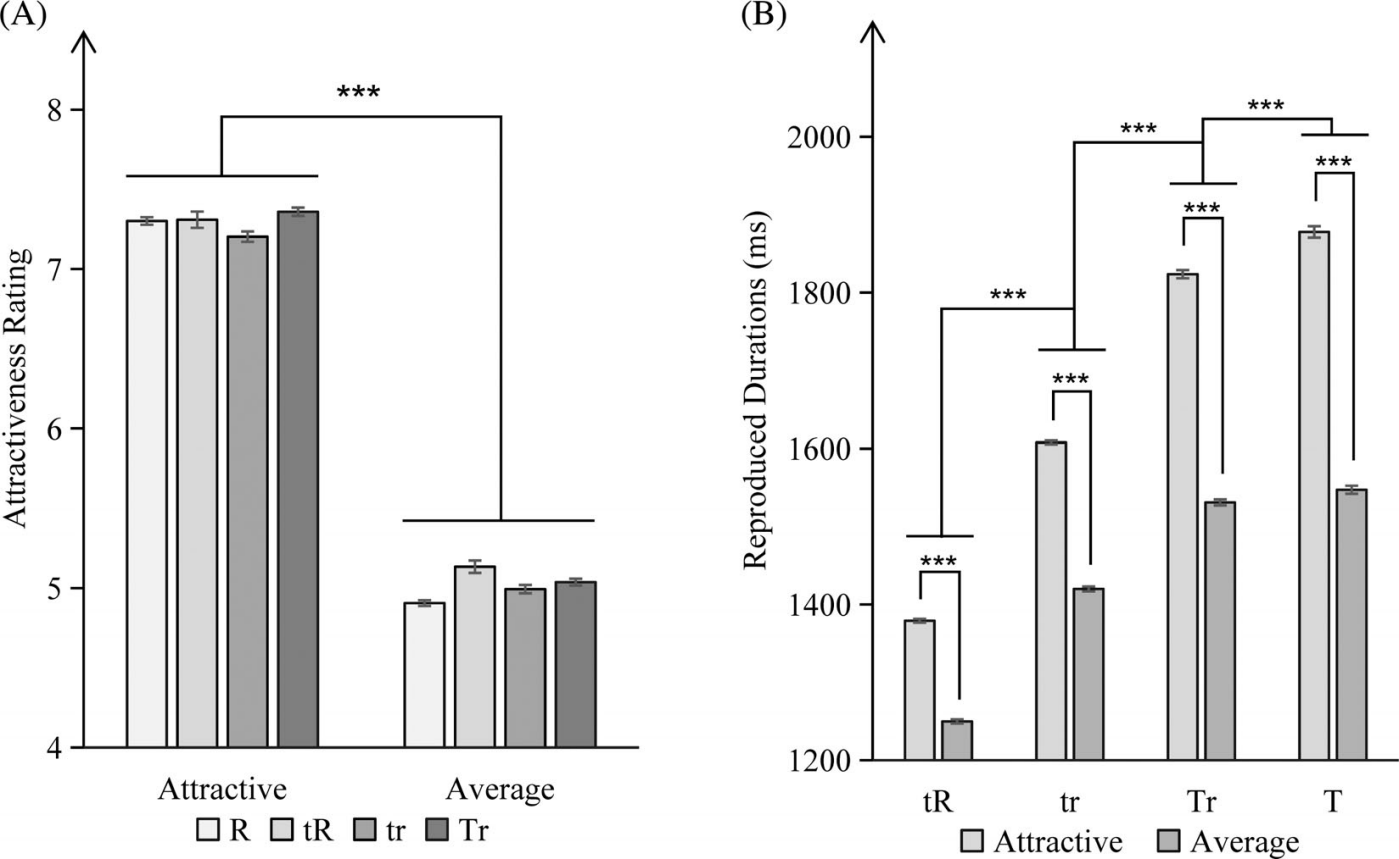
(A) Schematic representation of attractiveness rating for different stimulus type with different attention allocation. (B) Schematic representation of reproduced durations for different stimulus type with different attention allocation. Significance level ***p < .001.

In the attractiveness rating task, stimulus types (attractive, average) and attention allocation (R, tR, tr, Tr) were analyzed as within‐subjects factors in an ANOVA. A significant main effect of stimulus type was shown, *F*(1,39) = 15,950.25, *p* < .001, *η*_*p*_^2^ = .99. Post hoc tests (Bonferroni correction) suggested that the attractiveness ratings for a face that was appealing were consistently higher than those for average faces, irrespective of the allocation of attentional resources (*p* < .001). The findings are depicted in Figure 5A.

In the temporal reproduction task, the same ANOVA was taken. It was shown that the stimulus type had a significant main effect, *F*(1,39) = 5704.10, *p* < .001, *η*_*p*_^2^ = .99. It was shown in a post hoc test (Bonferroni correction) that the reproduction duration of attractive faces was consistently longer than that of average faces (*p* < .001). Additionally, there was a statistically significant main effect of attention allocation, *F*(3,37) = 2753.04, *p* < .001, *η*_*p*_^2^ = .99. Critically, a significant effect of interaction was found for type of stimulus and attention allocation, *F*(3,37) = 253.91, *p* < .001, *η*_*p*_^2^ = .95. The results are presented in Figure 5B.

Further comparisons revealed significant differences in the time dilation effect based on the allocation of attentional resources. Specifically, when average faces were presented, there was a stronger time dilation effect in the “T” condition than the “Tr” (*p* = .006). Additionally, the “Tr” condition exhibited a stronger time dilation effect than the “tr” (*p* < .001), while the “tr” condition showed significantly longer reproduced durations than the “tR” (*p* < .001). Similarly, when attractive faces were presented, allocating more attentional resources to the temporal task resulted in a significantly stronger time dilation effect (*p* < .001).

**FIGURE 6:**
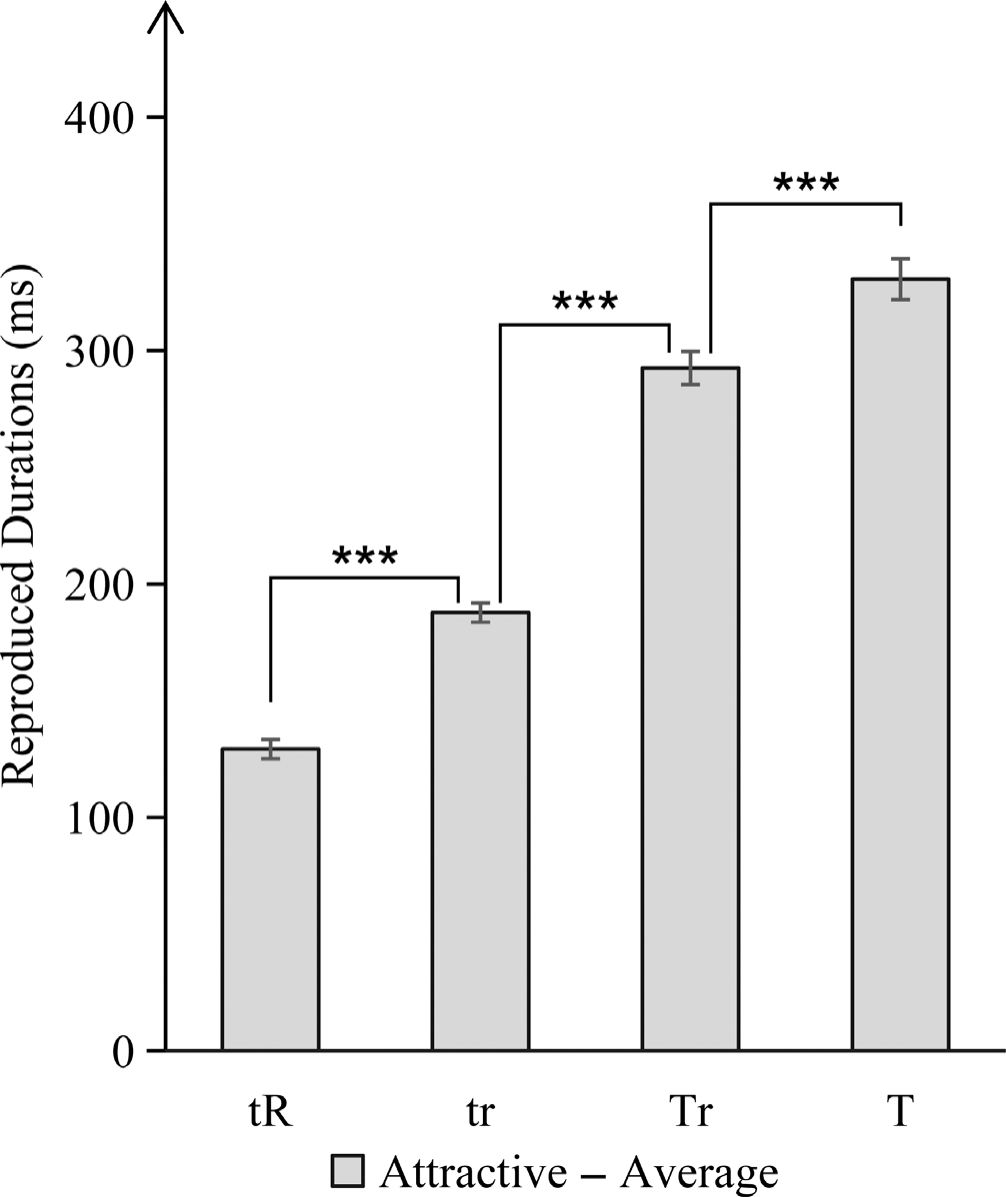
Schematic representation of the difference between the reproduced durations of attractive faces minus the reproduced durations of average faces with different attention allocation. Significance level ***p < .001.

To more directly observe how attention allocation affects the time dilation effect of facial attractiveness, the above findings were combined by subtracting the reproduced durations produced by attractive faces from the reproduced durations produced by average faces, and performing one‐sample *t*‐test on its difference value. It was found that under different attention‐allocation conditions, significant differences were found between all difference values (*p* < .001). The findings are displayed in Figure 6.

These results indicated that attention can manipulate time perception. In particular, during the process of facial attractiveness influencing time perception, regardless of whether attractive or average faces are presented, as long as more attentional resources were allocated to temporal task, it will result longer reproduced durations and will lead to an overestimation of time.

### Discussion

In Experiment 2, a dual‐task paradigm was adopted to study exactly what kind of connection there is between attention and time dilation effect. This paradigm involved two tasks: an attractiveness rating task and a reproduction task. By manipulating the allocation of attention between the two tasks, our goal was to exploit the connection for attentional processes with time dilation effect.

We began Experiment 2 by presenting attentional cues as a way to investigate the impact of facial attractiveness and attention on perceived time, and requiring participants to allocate attention to the time of stimulus presentation, or to facial attractiveness, or to both. The results showed that regardless of the allocation of attentional resources, attractive faces led to more significant time dilation effect compared to average faces, and the results of Experiment 2 were consistent with Hypothesis 2. There were also significant differences in resulting time dilation effect depending on the attentional resources allocated to the reproduction task.

On the basis of the observed results and previous research, it can be suggested that attention plays a crucial role within a time dilation effect of faces. The use of a dual‐task paradigm, which effectively adjusts the allocation of attention, has provided further evidence for this relationship (Chen et al., 2007; Hallez & Droit‐Volet, 2019). The findings suggested that attention performs an essential role in the time dilation effect produced by the face.

Based on existing findings, we calculated differences in the reproduced durations for each participant under both stimulus types and tested whether differences in attention allocation mediated the role of faces in influencing perception of timing. The findings demonstrated a significant mediating effect of attention on temporal dilation in both cases (appealing faces versus average faces). That is, increasing attention led to an enhanced effect of temporal dilation on faces, regardless of whether they were attractive or average faces, providing direct experimental evidence for the attentional mechanism behind the time dilation effect of facial attractiveness.

## GENERAL DISCUSSION

A primary objective of the research was to investigate the impact of attention on the perceived time produced by attractiveness of face. Earlier work has suggested that increased attention and arousal levels contribute to the overestimation of time perception (Lake et al., 2016; Piovesan et al., 2019; Vallet et al., 2019). The time dilation effect observed for attractive faces has been attributed to heightened arousal levels (Zhou et al., 2021). Although some scholars have argued that attention has a critical function within time dilation effect of facial attractiveness (Chen et al., 2007; Matthews & Meck, 2016), direct empirical evidence is lacking. To address this gap, Experiment 1 employed the dot‐probe task and temporal reproduction task to examine the mediating role of attention in the influence of facial attractiveness on perception of timing. Furthermore, Experiment 2 employed a dual‐task paradigm, incorporating an attractiveness rating task and a reproduction task, to study the association of attention allocation and time dilation.

Both Experiment 1 and Experiment 2 observed a time dilation effect associated with attractiveness of face. In Experiment 1, attractive faces elicited greater attention bias, resulting in a perception of longer durations compared to average faces, thus showing a significant time dilation effect. Similarly, Experiment 2 demonstrated that there were significant differences in reproduced durations for attractive faces and average faces, with attractive faces being reproduced longer. These findings align with prior research demonstrating that attractive faces can induce a dilation effect on perception of timing (Arantes et al., 2013; Ogden, 2013; Tian et al., 2019; Zhou et al., 2021). This implies that the impact of facial attractiveness on perception of timing is consistent, reliable, and stabilized. By successfully reproducing the time dilation phenomenon with the appealing face, the present study establishes a foundation for further investigations into the role of attention in this effect.

The findings of Experiment 1 support Hypothesis 1, which proposes that attention bias is a mediator of the time dilation effect of facial attractiveness. The findings demonstrate that attractive faces elicit a greater attention bias compared to average faces, indicating that highly attractive faces attract more attention, which is consistent with experience and previous research findings (Chen et al., 2012; Sui & Liu, 2009; Van Hooff et al., 2011). This attention bias, in turn, leads to a time dilation effect, where attractive faces are perceived as lasting longer than average faces. The attentional gate will open only when attentional resources are allocated to the temporal task, and allows for a higher number of pulses to be sent to the accumulator, resulting in a longer subjective time perception. Thus, several studies have theorized that enhanced attention is the main reason for time dilation effect of facial attractiveness (Ogden, 2013). Experiment 1 directly supports the inference: an increase in attention bias was observed to mediate a time dilation effect during the impact of facial attractiveness on perceived time.

Attractive faces capture more attention bias, which leads to a significant time dilation effect; this is because in the early stages of attention, individuals tend to be easily attracted to novel and salient stimuli and produce involuntary attention. In addition, in the middle and late stages of attention, attractive faces prompt individuals to develop tendencies and interests (Winston et al., 2007), which will attract more attentional resources. Therefore, to delve more thoroughly into the role of attention allocation in the impact of facial attractiveness on perceived time, Experiment 2 modeled the allocation of attention by using dual‐task paradigm, including attractiveness rating task and reproduction task. The dual‐task paradigm was applied to prove that temporal processing requires the engagement of attention (Bar‐Haim et al., 2010; Brown et al., 2013; Hallez & Droit‐Volet, 2019). On completion of the experiment, participants were guided to accomplish adherence ratings, and it was concluded from the results that the dual‐task paradigm was effective in motivating participants to allocate attentional resources as required by the task.

In Experiment 2, we hypothesized that the greater attention being distributed to time‐related tasks, the more it would enhance the impact of facial attractiveness on perception of timing. According to Hypothesis 2, our analysis of the role of attention in influencing time dilation effect found that, within attractive faces and average faces, an increase in attention to the temporal task significantly enhanced the time dilation effect of facial attractiveness. Many previous studies have indicated that subjective temporal intervals are prolonged when attention is allocated on temporal task, and shortened when attention is distributed to other tasks that are not related to time (Coull et al., 2004; Matthews & Meck, 2016). Similarly, this discovery is in line with earlier findings on the temporal expansion of emotions (Mella et al., 2011) that diminished time dilation is associated with a decrease in attentional resources used for timing. Furthermore, numerous studies have illustrated that appealing faces can influence lower‐level cognitive processes, such as attention (Chen et al., 2012; Lindell & Lindell, 2014; Maner et al., 2007; Sui & Liu, 2009). Attractive faces motivate individuals to allocate more attentional resources, resulting in a more significant time dilation effect. Faces influence time perception by capturing more attention and enhancing attention, to produce an overestimation of time intervals.

In summary, the findings of Experiment 1 and 2 consistently highlight that attention has an essential role in the impact of facial attractiveness on perceived time. Specifically, attractive faces elicit more attention bias compared to average faces, leading to an enhanced time dilation effect. From a biological evolutionary perspective, attractive faces elicit increased attention because they help individuals to select high‐quality mates and reproduce healthier offspring (Maner et al., 2007). As a result, this time dilation effect may serve to prepare individuals for potential courtship behavior rather than simply being a byproduct of heightened attention. However, the time dilation effect is enhanced when more attentional resources are allocated to the temporal task, regardless of whether they are average faces or attractive faces. We hypothesize that when attention is distributed to an assignment, it speeds up the cognitive process of the task and reduces the time it takes for it to enter consciousness (Huang & Qian, 2010), thus affecting time perception, that is, the task to be focused on can be perceived earlier, leading to the extension of time perception. Conversely, it has also been suggested that attention accelerates sensory processing and thus shortens perception; however, this is mainly caused by sensorial facilitating effects and modifications of attention to decision‐making mechanisms (Schneider & Bavelier, 2003). From another perspective, the influential role of attention in the time dilation effect also reflects the fact that time perception is highly flexible, with increased attention resulting in an overestimation of perceived time, and decreased attention leading to an underestimate regarding perception of timing. This flexibility in perceived time reflects the process by which individuals adapt to shifts in their environment (Cicchini et al., 2012; Kruijne & van Rijn, 2021; Lake et al., 2016).

Some limitations of the present research ought to be resolved in future studies. First of all, in the present research, the dot‐probe task was employed to detect attention bias and the dual‐task paradigm was utilized to manipulate the allocation of attention. However, the exploration of attention can rely not only on behavioral experiments, but also on some instruments to measure, such as some studies that have verified changes in attention caused by facial attractiveness through eye‐movement indicators (Valuch et al., 2015). Eye‐movement apparatus can further subdivide the process of attention, so that the role of attentional mechanisms in time dilation effect of facial attractiveness can be investigated in the future in terms of eye movements or other physiological indicators. Second, the participants in this study were all male. Previous studies have found similar behavioral performance of men and women towards attractive female faces, but the reasons behind this may be diverse and complex (Carrito et al., 2018; Tian et al., 2019). Therefore, this complex situation can be explored more deeply in the future. Third, since our selected participants were all heterosexual, and sexual minorities have also been a research hotspot in recent years, future studies could further examine whether there is a sexual orientation effect on the time dilation effect of facial attractiveness based on sexual minorities. Finally, in this study, we chose faces as the point at the center of the assessment of attractiveness. However, attractiveness is not only limited to the face, but the body torso, dress, and grooming all can produce attractiveness and are affected by cross‐cultural influences (Zhang et al., 2019). The reasons behind the time dilation effect triggered by attractiveness can be further explored in the future. Therefore, the impact of attraction on perceived time can be explored under diverse stimuli in the future to improve the stability of experimental results.

## CONCLUSIONS

The main intention of this study was to investigate the association for attention with the time dilation effect of facial attractiveness. The findings revealed that (1) the time dilation effect of facial attractiveness was mediated by enhanced attention bias triggered by facial attractiveness, and (2) a greater allocation of attention to the temporal task was correlated to a stronger time dilation effect of faces. Such findings indicate that increased attention serves as a crucial mechanism by which facial attractiveness leads to time dilation effect.

## CONFLICT OF INTEREST STATEMENT

All authors declare they have no conflicts of interest.

## ETHICS STATEMENT

This research was authorized by the Institute of Brain and Psychological Sciences, Sichuan Normal University, number SCNU‐20211221. This research was performed in accordance with appropriate ethical standards. All of the participants gave informed consent before participating in the study.
